# Learning Dynamics of Solitonic Optical Multichannel Neurons

**DOI:** 10.3390/biomimetics10100645

**Published:** 2025-09-24

**Authors:** Alessandro Bile, Arif Nabizada, Abraham Murad Hamza, Eugenio Fazio

**Affiliations:** Department of Fundamental and Applied Sciences for Engineering, Sapienza Università di Roma, 00161 Roma, Italy; arif.nabizada@uniroma1.it (A.N.);

**Keywords:** spatial solitons, solitonic logic gates, neuromorphic photonics, photonic neural networks, all-optical signal routing, nonlinear waveguide circuits

## Abstract

This study provides an in-depth analysis of the learning dynamics of multichannel optical neurons based on spatial solitons generated in lithium niobate crystals. Single-node and multi-node configurations with different topological complexities (3 × 3, 4 × 4, and 5 × 5) were compared, assessing how the number of channels, geometry, and optical parameters affect the speed and efficiency of learning. The simulations indicate that single-node neurons achieve the desired imbalance more rapidly and with lower energy expenditure, whereas multi-node structures require higher intensities and longer timescales, yet yield a greater variety of responses, more accurately reproducing the functional diversity of biological neural tissues. The results highlight how the plasticity of these devices can be entirely modulated through optical parameters, paving the way for fully optical photonic neuromorphic networks in which memory and computation are co-localized, with potential applications in on-chip learning, adaptive routing, and distributed decision-making.

## 1. Introduction

The human brain represents one of the most sophisticated computing systems known to nature [1]. It is capable of learning, adapting, and processing information in real time by leveraging a massively parallel network of interconnected neurons [2]. These neurons not only perform computation but also store information, thereby merging memory and processing into a single physical unit [3]. This structural and functional duality, enabled by synaptic plasticity, allows the brain to operate with exceptional efficiency, resilience, and adaptability. In contrast, traditional digital systems rely on separate units for processing and storage, connected through predefined interconnects and governed by fixed architectures. The resulting computational model—Von Neumann architecture [4]—suffers from significant limitations in speed, energy efficiency, and adaptability. To overcome these limitations, neuromorphic computing has emerged as a multidisciplinary approach aimed at designing hardware systems that replicate the functional principles of biological intelligence. Within this paradigm, neuromorphic photonics has gained considerable attention as a platform capable of achieving high-speed, low-energy, and parallel information processing.

Photonic neuromorphic computing has been actively explored through several technological platforms. In particular, Mach–Zehnder interferometer meshes have enabled large-scale linear transformations [5] phase-change materials have provided non-volatile synaptic weights [6,7], and microring resonators have been exploited to implement high-speed convolutional operations [8,9]. These diverse approaches highlight the breadth of the field and provide a useful benchmark against which our solitonic strategy can be compared. Recent developments have also extended to wetware-based architectures, in which biochemical systems themselves are harnessed as substrates for information processing. In such systems, molecular interactions, chemical oscillations, and reaction–diffusion dynamics are employed to realize computational tasks. These approaches, discussed in recent works [10,11], reveal unique features such as intrinsic self-organization, stochastic adaptability, and biochemical parallelism, properties that are extremely challenging to reproduce in solid-state media. In particular, wetware implementations naturally leverage the complexity and redundancy of chemical networks, offering emergent behaviors—such as adaptive learning or spontaneous reconfiguration—that transcend the deterministic framework of conventional hardware. The inclusion of wetware in the broader neuromorphic landscape thus highlights the diversity of possible implementations. Furthermore, it highlights the importance of complementary approaches, such as our photonic solitonic neurons, which offer stability, scalability, and high-speed processing.

Among the various photonic approaches, spatial optical solitons in nonlinear media offer a particularly promising avenue due to their ability to self-trap, self-adapt, and form stable reconfigurable interconnections [12]. Solitons are self-reinforcing wave packets that preserve their spatial and temporal profile during propagation, as a consequence of the delicate balance between dispersion and the nonlinear response of the medium. In optics, this phenomenon gives rise to spatial solitons, which behave as localized and robust beams of light capable of traveling long distances without diffraction. Such beams can effectively induce refractive index changes in the medium, thereby creating self-written and reconfigurable waveguiding channels. These features strongly resemble the behavior of biological synapses, where synaptic strength and pathway formation evolve based on the power and repetition of input stimuli. In recent research, we demonstrated how solitonic waveguides generated through photorefractive nonlinearity can mimic the dynamic reinforcement and weakening of synaptic connections [13], creating first solitonic neurons and then complex networks. These solitonic neural networks (SNNs) not only reproduce learning and forgetting mechanisms but also provide a structural basis for spatial memory and signal routing, paving the way toward truly biomimetic photonic hardware. While prior models have predominantly employed simple geometries such as X-junctions to implement basic neuron-like behavior, these structures propose a new class of multi-channel solitonic junctions capable of supporting higher-order computational functionalities. We now analyze and characterize their learning dynamics. These junctions extend the traditional two-beam interaction to multi-beam frameworks, enabling emergent behaviors such as optical decision-making, dynamic signal reconfiguration, and spatially modulated logic. By carefully engineering the angles, input configurations, and propagation paths of solitons, we demonstrate how these complex geometries can function as reconfigurable logic elements for neuromorphic systems. Moreover, soliton neurons show a learning mechanism similar to the biological one: when a synapse is not well localized, neurons not involved in learning a specific piece of information are also excited, leading to a loss of efficiency [14]. The formation of solitonic channels in a multichannel system also follows Hebb’s learning principle whereby “neighbouring neurons learn at the same time” [15]. Numerical simulations and experimental models validate the nonlinear interaction dynamics and the potential for learning-like adaptation through power-based channel reinforcement. This new architectural framework significantly expands the computing repertoire of solitonic systems, aligning them more closely with the biological counterpart. In doing so, it contributes a fundamental building block toward the realization of fully optical neuromorphic networks, in which information processing and memory formation are no longer sequential but spatially embedded and co-localized, as in the living brain. This article is constructed as follows. The first two paragraphs present a neurobiological description of some complex neuronal dynamics. The second part of the article, on the other hand, investigates solitonic structures, emphasizing how they are able to reproduce purely biological mechanisms. As far as we are concerned solitonic plasticity is the only hardware capable of analogically reproducing the tissue behavior of the biological neural system (other systems, like the ones based on PCMs give digital response which is far from the biological counterpart).

## 2. Synaptic Specificity, Hebbian Learning and Functional Heterogeneity

Effective learning in biological nervous systems requires that synaptic plasticity be confined to the connections directly involved in processing a given stimulus. This synaptic specificity is ensured by the spatial compartmentalization of intracellular signals—such as calcium, cAMP, and activated kinases—which prevents their diffusion to inactive synapses [16]. When such compartmentalization is lost, non-selective modifications occur: synapses unrelated to the stimulus undergo potentiation or depression, thereby reducing the accuracy of memory encoding [17]. Computational studies show that even modest levels of synaptic update diffusion degrade learning efficiency, reinforcing irrelevant connections and leading to a loss of fidelity in internal representations [18]. In summary, synaptic specificity constitutes an indispensable requirement for the stability of biological networks. From a photonic neuromorphic perspective, this principle implies that a soliton-based system must incorporate mechanisms to locally confine optical plasticity, preventing unintended refractive index changes from altering pathways not engaged in information processing [19,20]. Therefore, just as biological neurons rely on synaptic compartmentalization to prevent unwanted crosstalk, multichannel solitonic neurons confine plasticity through localized refractive index modifications, ensuring that only the stimulated pathways undergo effective learning. In biological neural networks, synaptic connections undergo a competitive process whereby the most efficient synapses are maintained and redundant or weak ones are eliminated. This phenomenon, known as synaptic collapse or pruning, is crucial for optimizing the efficiency and precision of neuronal transmission [21,22]. During development, many initial connections are progressively eliminated: for example, at the neuromuscular junction a single muscle is initially innervated by multiple motoneurons, but upon maturation only a single stable connection remains [23]. Similar processes occur in the visual system, where sensory experience guides the selection of inputs originating from one eye [24]. Selection is mediated by molecular and cellular mechanisms that discriminate between “strong” and “weak” synapses: calcium-dependent signaling, the availability of neurotrophic factors, and the activity of glial cells such as microglia, which identify and remove less active connections [25]. In analogy to synaptic pruning, where inefficient biological synapses are eliminated, solitonic networks reinforce the most efficient optical pathways while progressively suppressing less active ones, leading to an optimized distribution of energy across the structure. In optical neuromorphic terms, this principle suggests that multichannel solitonic neurons may also benefit from selection processes, strengthening the most efficient optical pathways while reducing or eliminating those that are less frequently engaged. Parameters such as writing power and temporal reiteration may play a role analogous to synaptic competition, enabling the photonic network to converge toward more stable and efficient configurations [26]. Hebb’s principle states that when two neurons are activated in a temporally correlated manner, the connection between them is strengthened [27]. This activity-dependent form of plasticity underlies processes such as long-term potentiation (LTP), in which the temporal correlation of pre- and postsynaptic stimuli durably enhances transmission efficacy [28]. In biology, Hebbian learning supports the formation of cell assemblies: groups of neurons that, through repeated co-activation, develop stable connections capable of partially reactivating one another (pattern completion) [29]. The key aspect lies in its associative nature: elements of experience that occur simultaneously or in close temporal sequence become linked within the network, forming coherent and retrievable representations [30]. The Hebbian principle, whereby coincident neural activations strengthen their connection, finds a direct photonic counterpart: when multiple coherent beams propagate simultaneously, their refractive index modulation favors those channels for future transmissions. In soliton-based optical neuromorphic systems, this principle can be translated into purely photonic terms: coherent and intense light beams simultaneously propagating along specific channels alter the refractive index of those channels, making them preferential for future propagations. The power and synchrony of optical stimuli thus play a role comparable to the frequency and correlation of neural impulses, enabling the photonic network to learn and consolidate optical configurations based on input history [31]. In biological nervous systems, structural diversity among neurons is tightly coupled with functional diversity. Pyramidal neurons, Purkinje cells, inhibitory interneurons, and motoneurons—although all sharing the fundamental property of electrical excitability—exhibit radically different morphologies, synaptic densities, and connectivity patterns, each optimized for specific tasks [32,33]. This heterogeneity is not accidental; rather, it is the result of functional specialization that enables networks to integrate sensory inputs, perform complex computations, modulate signals, and generate targeted outputs [34]. The distribution and roles of these neuronal types determine the balance between response speed, accuracy, memory capacity, and long-term stability [35]. For example, neurons with extensive dendritic arborizations can integrate signals across wide cortical areas, whereas rapidly projecting neurons maximize transmission speed at the expense of spatial integration [36]. This principle of diversification is directly transferable to photonic neuromorphic systems. In solitonic optical neurons, varying the number of channels and nodes produces analogous behaviors: some architectures favor rapid learning and high energy efficiency, while more complex ones sacrifice speed in order to achieve a broader range of states and richer response patterns. As in neural tissue, the coexistence of optical units with distinct properties enhances the overall capacity of the network to solve complex tasks, integrating “fast-response” modules with “extended-memory” modules [37]. Biological tissues exploit neuronal diversity—pyramidal cells, interneurons, Purkinje cells—to balance speed, memory, and precision. Similarly, the coexistence of single-node and multi-node solitonic neurons provides complementary functionalities: the former offering rapid, energy-efficient learning, and the latter delivering richer dynamical responses and functional diversity.

## 3. Optical Solitons and Simulation Method

Photorefractive crystals are semiconductors that exhibit a second-order electro-optic nonlinearity, which allows their refractive index to vary under an externally applied static electric field. The photorefractive nonlinearity originates from the electro-optic effect, namely the modification of a material’s refractive index under the influence of a static electric field. Photorefractive materials are usually semiconductors that can absorb photons through electronic transitions from trap states into the conduction band. As a result, light absorption produces two types of charge carriers: electrons that are free to move within the conduction band and holes that remain bound in trap states. The generation of free electrons and localized holes gives rise to a local electric field, which modifies the refractive index of the photorefractive material through the electro-optic effect. Since the charge distributions are spatially localized, the resulting photoinduced field—and consequently the refractive index change—is also confined to specific regions [38]. When a Gaussian laser beam is focused into a photorefractive medium, its bell-shaped power profile produces a refractive index modulation resembling a microlens, capable of refocusing the light. Extending this mechanism across the medium allows the formation of narrow, high-index channels that act as optical waveguides, effectively trapping light within them. The refractive indices obtain the expressions:

(1) $$\left\{\begin{array}{c} n_{x}=n_{y}=n_{o}\\ n_{z}=n_{e}-\frac{1}{2}n_{e}^{3}r_{33}(E_{bias}+E_{sc}) \end{array}\right.$$

where *E_bias_* is the vectorial bias electric field, *E_sc_* is the screening electric field produced by light propagation, and *n_o_* and *n_e_* are, respectively, the ordinary and the extraordinary refractive indices. This phenomenon corresponds to the creation of a spatial soliton, a self-confined beam guided by the refractive index variation it induces. Importantly, the induced index change is a genuine material modification and can guide other wavelengths as well, enabling soliton-written channels to function as waveguides for additional signals. We consider lithium niobate (LiNbO_3_) crystals for which optical channels are typically written parallel to the crystallographic c-axis, in order to maximize the nonlinear response through the electro-optic coefficient r_33_. This orientation enhances the self-trapping of beams and facilitates the formation of spatial solitons, which act as reconfigurable light-guiding channels. The simulations were performed by using a previously validated FDTD numerical code. The model solves the nonlinear propagation equations under the Slow Varying Envelope Approximation (SVEA). The underlying operational principle consists of 2 phases corresponding, respectively, to the writing phase of the neural structure and to the learning phase. During the writing phase, a variable number n of light beams with hyperbolic secant profiles (*A_i_*) are launched into a nonlinear lithium niobate crystal. At this stage, the beams are equal in power, thereby inducing identical refractive index variations. They propagate following the Helmholtz equation with saturating electro-optic nonlinearity:

(2) $${𝛻}^{2}A_{i}=-\;\frac{\varepsilon_{NL}E_{bias}}{1+\frac{|{\sum_{i=1}^{n}A_{i}|}^{2}}{{\left|A_{sat}\right|}^{2}}}\;A_{i}$$

where *ε_NL_* is the nonlinear dielectric constant, *E_bias_* is the bias electric field that can be activated by electrodes or by pyroelectric effect [39] but it is necessary for screening solitons, ${\left|A_{sat}\right|}^{2}$ is the saturation power. All the light writing beams can induce a nonlinear response in the material while the signal light beams A_n+1_ feels only the modification without modifying it. The electrostatic bias has been set at 40 kV/cm, while the light powers at 10 μW for the writing beams (at the input face) and at 0.5 μW for the signal one. Once the structure is established, it becomes possible to inject a signal at a different wavelength (A_n+1_), to which lithium niobate is not photosensitive. This signal propagates through the channels in a manner analogous to waveguide transmission. However, when it reaches overlap regions between channels, it begins to experience local variations in the refractive index profile and accordingly splits among the available branches. Thus, in configurations where the overlapping channels are characterized by equivalent index modulations, the signal is evenly distributed across the output channels. This behavior holds as long as the mutual writing angle between solitonic channels does not deviate significantly from 1°. At the end of the writing phase, all multichannel solitonic neurons are thus balanced. This balanced refractive index map constitutes the starting point for the learning process. The learning of the neuron can occur either in a supervised or unsupervised fashion. In both cases, learning proceeds through the temporal evolution of the refractive index. The continued injection of writing light induces further variations, which can either be externally controlled—as in supervised learning—or governed by internal mechanisms, as in unsupervised learning. In both scenarios, the neuron eventually becomes unbalanced, evolving toward a configuration shaped by the processed and stored information. Solitonic channels demonstrate a capacity for self-regulation in information routing, effectively suppressing weak connections in favor of stronger ones. This process is entirely light-driven and reflects two key biological principles:

- If a channel is written or rewritten with significantly higher beam power than the others, it experiences a more pronounced local index variation. As a result, weaker channels tend to merge into the stronger one and disappear.
- If the writing beam remains in a given channel for a longer duration than in others, the deeper index modulation ultimately causes the collapse of the competing channels.

## 4. Multichannel Solitonic Neurons Learning

The ability of spatial solitons to establish plastic connections has already been demonstrated in previous works [37]. More recently, it has been shown that the dynamics underlying soliton formation are governed by mathematical principles that closely resemble those involved in the development and adaptation of biological synaptic connections. This parallelism endows photorefractive systems with the potential to construct truly intelligent optical fabrics, where synaptic density is not shaped by the migration of neurotransmitters but rather by charge transport. In both systems, connections emerge or are modified in response to received signals [30]. The X-junction photonic neuron, formed through the intersection of two solitonic channels, can receive, processing, and storing information [26]. Recently, we have proposed new neuron configurations that are no longer defined by a pair of channels but by a number *n* = 3, 4, 5. It is therefore essential to understand how multichannel neurons are able to receive and process information in order to integrate them with the solitonic technology already developed. Therefore, in the present study, we explored the learning properties of multi-channel neural photonic units in lithium niobate crystals. The different learning dynamics are a fundamental characteristic of neural complexity. The physics governing the operation remains the same as that of a basic X-junction. The learning dynamics instead depend on the number of channels and nodes. Each multichannel neuron exhibits a specific learning dynamic, characterized by variations in both the timescales of imbalance and the attainable states. In general, complete learning requires longer times as the number of channels increases. However, it is important to emphasize that these times differ significantly between multi-node and single-node structures. The primary reason lies in the fact that multi-node architectures repeatedly partition the available energy, reducing it at each successive node. For this reason, even within the same neuron typology, certain channels reach imbalance more rapidly, while others require higher intensities or longer durations. The outcome depends on the number of nodes encountered during propagation: the greater the number of nodes, the more times the information-associated energy is subdivided, thereby necessitating either extended timescales or increased power.

### 4.1. Single-Node Neurons

**Figure 1:**
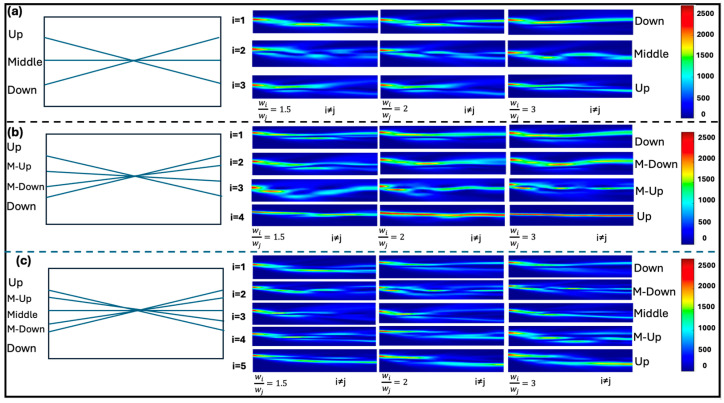
Learning evolution in single-node multichannel neurons (3 × 3, 4 × 4, 5 × 5) as a function of the power ratio between the trained input channel (*w_i_*) and the others (*w_j_*). Signal injection in the UP channel progressively establishes preferential pathways with increasing relative power. The cases of single-node neurons 3 × 3 (**a**), 4 × 4 (**b**) and 5 × 5 (**c**) are shown respectively.

In this class of neurons, structural symmetry generally prevails. The 3 × 3 neuron, for instance, constitutes an X-junction, with a central channel that divides the geometry into two equal portions. The 4 × 4 neuron, by contrast, distributes two pairs of channels symmetrically with respect to the axis bisecting the crystal along the propagation length. The 5 × 5 neuron follows the same logic as the 3 × 3 configuration but incorporates two additional channels—one on each side—relative to its “smaller sibling.” For this reason, and without loss of generality, we report the learning results of 3-, 4-, and 5-channel neurons as a function of the writing power employed, considering only the case of signal injection in the upper channel, which can be identified by referring to the schematic representations of the neurons in Figure 1. The imbalance primarily depends on the local modification of the refractive index, which may be induced either by the intensities of the writing beams or by their reiteration along a specific path.

Figure 1 clearly shows that multichannel neurons are characterized by a topological evolution dependent on the information being processed: if the information is important, it is encoded through a more intense signal. The input channel representing that information undergoes a more pronounced refractive index variation, thereby privileging its output. By examining each column of the three single-node multichannel neuron models, one can observe the propagations of signals injected into the upper channel (UP channel), as the channels highlighted by stronger index contrasts progressively change. For each structure, three different power ratios between the selected channel and the others were investigated. *w_i_* and *w*_j_ represent the input intensities of the beams: *w_i_* is related to the higher power channel, while *w_j_* is related to the other channels (i.e., i is always different from j). The learning dynamics depend on the ratio between the input intensities.

At a ratio of 1.5, the signal is not yet well confined to preferential directions and begins to split into different amounts across the various outputs. Increasing the ratio to a factor of 2 reveals that, in almost all configurations, the privileged behavior of the selected channel becomes predominant. When the ratio reaches a factor of 3, the power differences are pronounced, the refractive index defines a well-marked propagation path, and the information flows in a highly confined manner. Figure 1 also highlights another crucial aspect of learning. Neurons with an odd number of channels achieve a lower imbalance of the trained channel compared to neurons with an even number of channels (e.g., 2 × 2 [31] and 4 × 4). This effect is likely attributable to the presence of a channel parallel to the propagation direction, which hinders the redistribution of optical energy throughout the structure. In line with this observation, our previous study demonstrated that the presence of “central” channels increases the likelihood of solitonic channel collapse toward themselves. Such a channel therefore acts as an attractor for its neighbors. However, when parameters do not permit this collapse, the structure remains stable, though the central channel continues to function as an energetic attractor. In this framework, the notion of an energetic attractor refers to a stable state of the multichannel solitonic system, determined by the balance of nonlinear interactions. Such states can be interpreted as local minima of an effective energy functional, representing configurations toward which the system spontaneously evolves. In analogy with neural computation, these attractors act as robust memory states, ensuring reproducibility and stability in the processing of information. Moreover, what Figure 1 makes clear is that when one channel is strengthened, the immediately adjacent channel also increases its guiding strength—provided the two are co-active—albeit to a lesser extent. The same correlation-dependent reinforcement is observed in multichannel multi-node neurons co-activated adjacent channels are strengthened together because their optical fields and space-charge distributions partially overlap, increasing the local refractive-index change (Δn). Interpreting Δn (or the effective guiding contrast) as a synaptic weight, this corresponds to a Hebbian mechanism—“channels that fire together, wire together”—where simultaneous activity leads to selective, local reinforcement, whereas inactive or uncorrelated channels do not gain strength (and may even decay with time/iterations)

**Figure 2:**
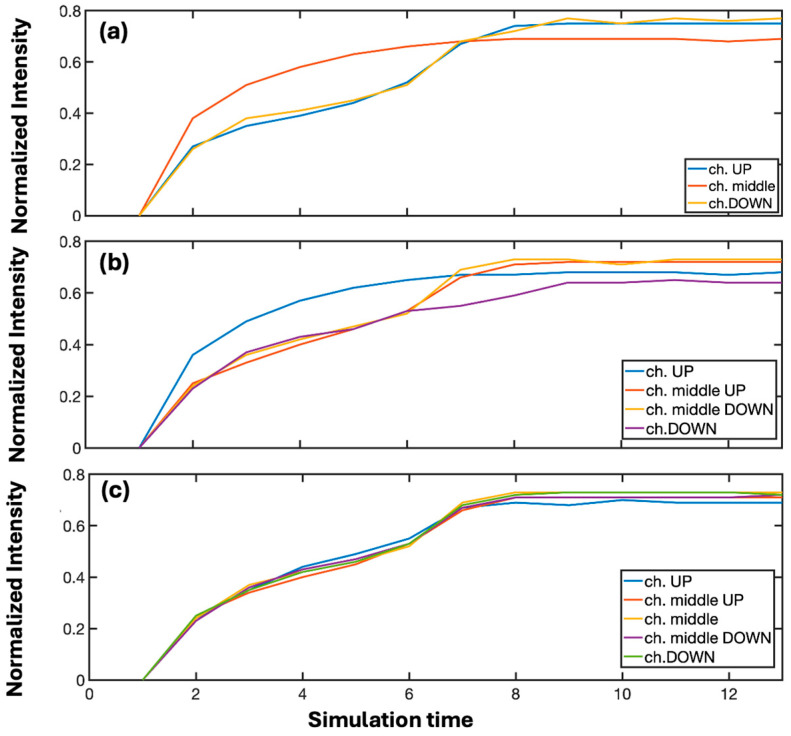
Level of imbalance in the trained channel of single-node neurons as a function of the number of light reiteration cycles. The final value is compared with that obtained for a power ratio of 3, demonstrating the convergence of the two learning dynamics. The cases of single-node neurons 3 × 3 (**a**), 4 × 4 (**b**) and 5 × 5 (**c**) are shown respectively.

As a result, the degree of imbalance among the channels remains lower. Everyday experience, later confirmed by neuroscience, has long taught us that *repetita iuvant.* When a signal is repeatedly presented in time, it persists in the brain by establishing strong and lasting synaptic connections. Repetition thus becomes synonymous with information that must be memorized—just as high power signals correspond to information that must be instantly encoded for the survival of the individual. In fact, the ultimate effect of reiteration corresponds to that of data with a high informational content. Figure 2 reports the level of imbalance in the trained channel as a function of the cycles of light reiteration, up to the same imbalance level reached when the beam intensities differ by a factor of 3. The *x*-axis does not correspond to a physical time scale. Instead, it indicates the number of discrete iterations performed during the numerical integration of the governing equations. Each iteration can be regarded as a computational step of the algorithm, allowing us to track the convergence of the system toward stable multichannel configurations.

We also observed a minor geometric component influencing the degree of imbalance as a function of the input signal position. This indicates that while the signal is affected by local refractive index modifications, it achieves slightly greater imbalance when it follows a geometrically favorable direction than when it propagates along a less congenial one. Taking the 3 × 3 structure shown in Figure 1 as an example, one can observe that when the signal is injected into the UP channel, the corresponding DOWN output exhibits a higher imbalance; conversely, when the signal is injected into a DOWN channel, it is the UP output that displays a stronger imbalance.

### 4.2. Multi-Node Neurons

**Figure 3:**
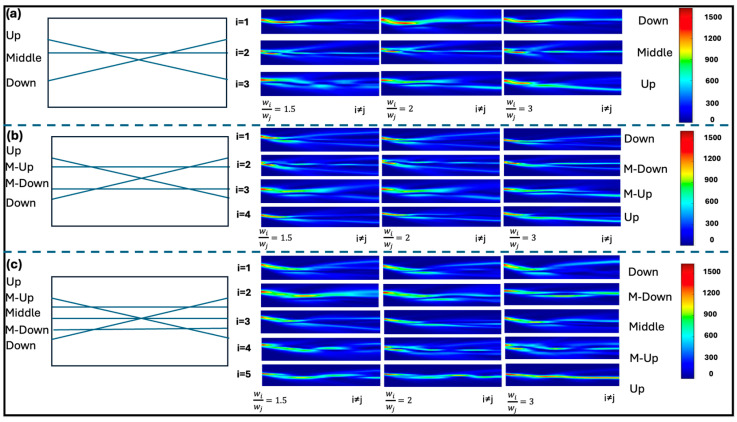
Comparison of learning in 3 × 3, 4 × 4, and 5 × 5 multi-node neurons for a signal injected into the UP channel as a function of the power ratio between the trained input channel (*w_i_*) and the others (*w_j_*). Propagation maps illustrate the formation of preferential optical pathways and the reduction in maximum imbalance levels compared to the corresponding single-node structures. The cases of multi-nodes neurons 3 × 3 (**a**), 4 × 4 (**b**) and 5 × 5 (**c**) are shown respectively.

**Figure 4:**
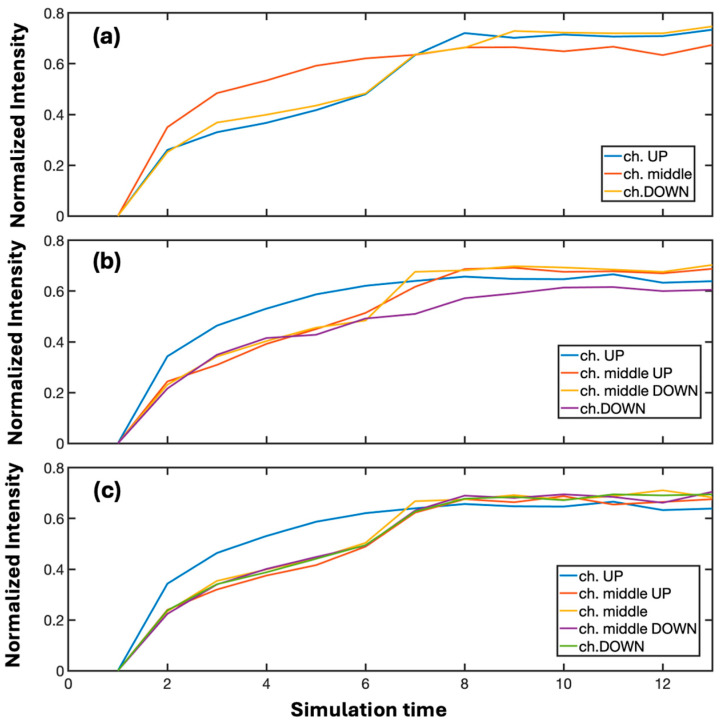
Dependence of the final imbalance in multi-node neurons on the number of channels and nodes. The trend reveals a nonlinear reduction in learning capacity with increasing geometrical complexity. The cases of multi-nodes neurons 3 × 3 (**a**), 4 × 4 (**b**) and 5 × 5 (**c**) are shown respectively.

Multi-node multichannel neurons generally exhibit a highly symmetric structure, with the exception of the 3 × 3 configuration. This class of neurons is constructed starting from the X-junction, to which additional channels are progressively added parallel to the axis of the crystal. The 3 × 3 multi-node neuron, in particular, is markedly asymmetric because the third channel is added either above or below the crystal’s symmetry axis (corresponding to the direction of light propagation). However, the two configurations are structurally and functionally equivalent, both in terms of their implementation and their learning dynamics, as illustrated in Figure 3. For multi-node neurons, we have likewise investigated the evolution of learning as a function of input intensities (Figure 3) and temporal reiteration (Figure 4).

Considering Figure 3, where 3-, 4-, and 5-channel multi-node neurons are required to learn information injected into the UP channel, the most evident aspect is that the final imbalance levels are almost always lower than those obtained with the corresponding single-node neurons. Specifically, the difference factor ranges between 1.35% and 7.66%, reaching values that are far from negligible. In biological systems, however, not all neurons exhibit the same properties of plasticity and signal transmission. From the perspective of achieving a fully photonic neuromorphic hardware capable of replicating the characteristics of neural tissue, the availability of neuron typologies that differ so markedly from one another is fundamental, as it ensures a high degree of functional complexity.

**Figure 5:**
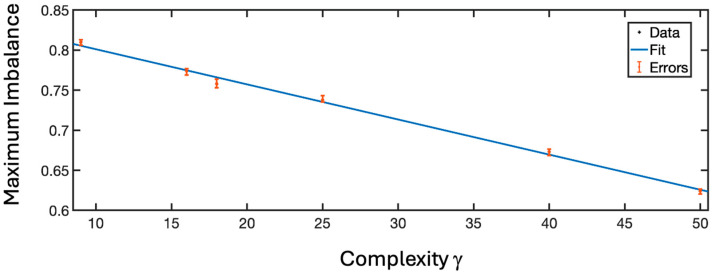
Nonlinear law of imbalance reduction in multi-node neurons as a function the complexity γ.

When discussing multi-node neurons, it is also important to note that increasing complexity (i.e., the number of channels and, consequently, of nodes) leads to a decrease in imbalance capacity. Accordingly, the 4 × 4 neuron exhibits a much lower imbalance than the 3 × 3 neuron, but only slightly higher than the 5 × 5 configuration. We have therefore theorized a parameter to indicate the level of complexity described by Equation (3):

(3) $$\gamma =\;n_{nodes}\cdot \;n_{ch}\cdot n_{ch\_cnode}$$

where $n_{nodes}$ is the number of nodes, $n_{ch}$ is the number of channels and $n_{ch\_cnode}$ is the number of channels passing by the central node, which acts as a general signal sorting center. Figure 5 represents the maximum imbalance of the reinforced channels as a function of the level of complexity of the structure. This linear behavior has a slope of −0.044 and a y-intercept of 0.84 (with a confidence of the R^2^ test of 0.985).

## 5. Conclusions

This work presents, for the first time, a detailed comparative analysis of the learning dynamics in multichannel solitonic optical neurons, both single-node and multi-node, highlighting how structural design profoundly influences performance. Simulations and observations demonstrate that single-node neurons ensure faster learning times and high energy efficiency, owing to their ability to reorganize optical pathways with minimal power variations. By contrast, multi-node neurons, while requiring higher intensities and longer times to reach imbalance, exhibit more articulated behavior and a broader variety of responses, more faithfully replicating the functional diversity typical of biological neural tissues. From a photonics perspective, this structural diversification demonstrates the feasibility of designing adaptive optical neuromorphic circuits in which memory and information processing are co-localized within the same physical substrate, thereby mitigating the bottlenecks inherent in von Neumann architectures. The ability to modulate the degree of plasticity through purely optical parameters—such as writing power, number of nodes, and temporal reiteration—opens promising avenues for the development of full-optical systems capable of on-chip learning, distributed decision-making, and real-time reconfiguration. From a neuromorphic engineering standpoint, the integration of neurons with different learning speeds and modalities enables the construction of more complex networks capable of balancing rapid adaptation with long-term stability, closely mirroring biological systems. These results represent a fundamental step toward the realization of cognitive photonic platforms, where light not only transports information but also processes, stores, and structures it according to neurobiologically inspired principles. The relevance of the biological analogy in our approach does not lie in a direct, one-to-one correspondence between biochemical and photonic mechanisms, but rather in the functional properties that emerge at the network level. In particular, two aspects are worth emphasizing. First, the solitonic neuron model allows for the coexistence of heterogeneous neuronal units depending on the number of nodes and channels that, despite their structural differences, can remain functionally interoperable. This reflects a fundamental property of biological neural systems, where neurons of different morphologies and connection patterns contribute jointly to information processing. Second, our system exhibits the ability to create and reshape dynamic channels as a function of the input signals thanks to charge motion. These channels do not merely redistribute energy passively; rather, they adaptively reconfigure the guiding structures of the network. In this way, signals sculpt the architecture itself: first by shaping the behavior of individual solitonic units, and subsequently by defining the connectivity of larger ensembles. Such plasticity resonates with the organizational principles of biological neural networks, where external stimuli continuously remodel both local synaptic strengths and the global topology of circuits by activating the neurotransmitter motion. By highlighting these two levels—heterogeneity and dynamic channel formation—we establish a functional bridge between photonic solitonic neurons and the adaptive complexity of biological neural computation.

## Data Availability

The data presented in this study are available on request from the corresponding author.
